# Longitudinal relationships between sub‐clinical depression, sub‐clinical eating disorders and health‐related quality of life in early adolescence

**DOI:** 10.1002/eat.23928

**Published:** 2023-03-09

**Authors:** Bridget Kenny, Steven J. Bowe, C. Barr Taylor, Marj Moodie, Vicki Brown, Elizabeth Hoban, Joanne Williams

**Affiliations:** ^1^ School of Health and Social Development, Faculty of Health Deakin University Geelong Victoria Australia; ^2^ Biostatistics Unit, Faculty of Health Deakin University Geelong Victoria Australia; ^3^ School of Medicine, Department of Psychiatry and Behavioral Sciences Stanford University Stanford California USA; ^4^ Center for m2Health Palo Alto University Palo Alto California USA; ^5^ Deakin Health Economics, School of Health and Social Development, Faculty of Health Deakin University Geelong Victoria Australia; ^6^ School of Health Sciences Swinburne University of Technology Hawthorn Victoria Australia

**Keywords:** adolescence, bi‐directional, depression, eating disorders, health‐related quality of life, longitudinal

## Abstract

**Objective:**

A comprehensive understanding of the relationship between depressive symptoms and eating disorder (ED) symptoms requires consideration of additional variables that may influence this relationship. Health‐related quality of life (HRQOL) has been associated with both depression and EDs; however, there is limited evidence to demonstrate how all three variables interact over time. This study sought to explore the bi‐directional relationships between depressive symptoms, ED symptoms and HRQOL in a large community sample of young adolescents

**Method:**

Adolescents (*N* = 1393) aged between 11 and 14 years (*M* = 12.50, *SD* = 0.38) completed an online survey measuring depressive symptoms, ED symptoms and HRQOL. Two‐level autoregressive cross‐lagged models with three variables (i.e., depressive symptoms, HRQOL and ED) assessed across two time points (T1 and T2) were created to address the study aims.

**Results:**

HRQOL was found to predict depressive symptoms and depressive symptoms were found to predict ED symptoms. Components of HRQOL (i.e., social relationships and ability to cope) were found to share a differential relationship with depressive symptoms. Inability to cope predicted depressive symptoms and depressive symptoms predicted negative social relationships. EDs were found to predict reduced HRQOL and negative social relationships.

**Discussion:**

Findings suggest prevention and early intervention programs designed to address adolescent depression should focus on improving HRQOL. Future research should assess the relationship between HRQOL and individual ED symptoms (e.g., body‐related ED symptoms, restrictive symptoms) as a means of exploring relationships that may have been masked by assessing ED symptoms using a total score.

**Public Significance:**

This study sought to explore how eating disorders, depressive symptoms, and health‐related quality of life (HRQOL) relate over time in a sample of young adolescents. Findings indicate adolescents who self‐reported lower levels of HRQOL, including a reduced ability to cope, are at risk of experiencing depressive symptoms. Adolescents should be provided with the tools to develop problem‐focused coping strategies as a means of reducing depressive symptoms.

## 1 INTRODUCTION

Eating disorders (EDs) are characterized by disturbances in behaviors and cognitions pertaining to food, eating and body image (American Psychiatric Association, 2013). They often emerge during adolescence (Volpe et al., 2016) and can have a profound impact on an individual and their wider family unit (Schaumberg et al., 2017). EDs, full threshold and sub‐clinical disorders (i.e., ED symptoms that do not meet full diagnostic criteria), are associated with psychological distress and increased risk of premature death as a result of medical complications and suicide (Karkkainen et al., 2017; Saeedzadeh Sardahaee et al., 2017; Schaumberg et al., 2017). EDs are often comorbid with other psychiatric conditions including depression (Goldschmidt et al., 2016; Hughes et al., 2013; Touchette et al., 2011).

Depression is a common mental disorder characterized by persistent feelings of sadness and loss of interest in daily activities (American Psychiatric Association, 2013). Depression often emerges during adolescence (Thapar et al., 2012) and, during this developmental period, it is a significant predictor of depression in adulthood (Johnson et al., 2018). Adolescents who experience depression report significant impacts on their functioning and interpersonal relationships (Lawrence et al., 2015) and are at increased risk of suicide ideation (Im et al., 2017).

EDs with a comorbid diagnosis of depression are associated with more severe symptoms and worse treatment outcomes (Hughes et al., 2013). As such, understanding the developmental trajectories of these disorders, and the relationship they share, is of public health importance. This is particularly true in the context of sub‐clinical symptomatology. Sub‐clinical EDs and sub‐clinical depression are highly prevalent in adolescence. It estimated that been 15% and 61% of adolescents experience ED symptoms (Koushiou et al., 2019; Neumark‐Sztainer et al., 2011; Sparti et al., 2019) and between 17% and 34% experience sub‐clinical depression (Crockett et al., 2020; Shorey et al., 2022; Tang et al., 2019). Understanding how depression and EDs relate at a sub‐clinical level at this crucial developmental stage may aid in the design and implementation of prevention and early intervention strategies to reduce the onset of disorder predictive symptomatology (Schaumberg et al., 2017). Research demonstrates that early detection and intervention of depression and EDs are likely to improve treatment outcomes (Schaumberg et al., 2017).

A comprehensive understanding of the relationship between depression and EDs requires consideration of additional variables that may impact these variables overtime. Health‐related quality of life (HRQOL) has been associated with depression (e.g., Freire & Ferreira, 2018; Shin et al., 2022) and EDs (Jalali‐Farahani et al., 2015; Zervaki et al., 2017) and therefore may influence the development of depressive symptoms and ED symptoms. HRQOL is a multidimensional concept that assesses an individual's perception of their position in life (Revicki et al., 2014). In contrast to measures of psychological distress, HRQOL considers a broad range of domains (e.g., physical, psychological and social) that may influence an individual's wellbeing (Jenkins et al., 2014). It is suggested that adolescents who experience defects in one or more of these domains may be more susceptible to experiencing an increase in depressive symptoms. Given depressive symptoms have been identified as a significant predictor of ED symptoms (Kenny et al., 2022; McCabe & Ricciardelli, 2006; Sander et al., 2021) an increase in depression symptoms, as a result of reduced HRQOL, may subsequently lead to an increase in ED thoughts and behaviors. Understanding how adolescents' perception of their lives (i.e., HRQOL) influences their experience of depressive symptoms and ED symptoms, and vice versa, can provide insight into the development and impact of sub‐clinical symptomatology. This information may be used to inform prevention and early intervention approaches.

It is suggested that aspects of HRQOL (e.g., social relationships, ability to cope) are more relevant to adolescent populations and consequently may be particularly important when exploring the relationship between depression symptoms, HRQOL and EDs during this developmental period. Adolescence embodies the critical transition between childhood and adulthood. During this period, adolescents often feel pressure to develop their personal identity while navigating changing social relationships and increased academic demands (Hellström & Beckman, 2021); this can be overwhelming and may impact their ability to cope. Furthermore, social interactions are increasingly important during adolescence and there is a strong desire to feel socially accepted and connected to their peers (Long et al., 2020; Orben et al., 2020). A failure to form strong social bonds within peer groups can adversely impact adolescents mental health (Long et al., 2020).

Despite evidence demonstrating associations between HRQOL and depression (e.g., Freire & Ferreira, 2018; Shin et al., 2022) and HRQOL and EDs (Jalali‐Farahani et al., 2015; Mitchell & Steele, 2017; Zervaki et al., 2017), to the authors' knowledge, few prior studies have investigated how all three variables interact over time in non‐clinical samples of young adolescents. To address this gap within the current evidence base, this study sought to explore the temporal relationships between depression, EDs and HRQOL among community samples of young adolescents. Specifically, this exploratory study aimed to explore (1) the bi‐directional associations between depressive symptoms, ED symptoms and HRQOL; (2) the bi‐directional associations between depressive symptoms, ED symptoms and the social relationships component of HRQOL; and (3) the bi‐directional associations between depressive symptoms, ED symptoms and the ability to cope component of HRQOL. Given female and male adolescents may experience depression and EDs differently (Kinasz et al., 2016; McGuinness et al., 2012), a secondary aim of this study was to explore these associations separately for females and males. Due to the lack of prior research in this area, no specific hypotheses were identified.

## 2 METHODS

### 2.1 Participants

The sample (*N* = 1393) included adolescents (629 females, 705 males, 59 did not disclose sex) participating in the Supporting Healthy Image, Nutrition and Exercise (SHINE) study. The SHINE study is a cluster randomized controlled trial designed to test the effectiveness of a tailored web‐based body image and weight management program for secondary school students in Victoria, Australia. Students enrolled in Year 7 (i.e., first year of secondary school) at 12 secondary schools (7 = independent and 5 = government) in metropolitan Melbourne (*N* = 8) and regional Victoria (*N* = 4) completed an online questionnaire between February and July 2019 (T1) and again 12 months later (February to March 2020, T2). At baseline 108 (8%) students did not complete the survey and at T2 245 (18%) did not complete the survey; this did not differ by age, sex, baseline depressive symptoms or baseline ED symptoms.

All data collection occurred prior to the COVID‐19 pandemic. The web‐based intervention was delivered within 2 weeks of baseline data collection. Preliminary analysis found the web‐based intervention did not have a significant impact on depressive symptoms or ED symptoms at T2. Participants were aged between 11 and 14 years (*M* = 12.50, *SD* = 0.38) at baseline. The Index of Relative Socio‐economic Disadvantage (IRSD) (Australian Bureau of Statistics, 2011), based on participants residential postal area code, was used to assess the relative disadvantage of the sample. IRSD incorporates information about the economic and social conditions of an area and provides a score from 1 to 10 where 1 is relatively greater disadvantage and 10 is relative lack of disadvantage (Australian Bureau of Statistics, 2011). IRSD scores indicated relatively low levels of socio‐economic disadvantage with 9% of adolescents in the first (i.e., lowest) quintile based on Australian population norms, 15% in the second quintile, 15% in the third quintile, 20% in the fourth quintile and 41% in the fifth (i.e., highest) quintile.

### 2.2 Measures

#### 2.2.1 Eating disorder symptoms

The Eating Disorder Examination‐Questionnaire‐Adolescent version (EDE‐A) (Carter et al., 2001; Fairburn, 2008) is a 36‐item self‐report measure of ED symptoms over the past 14 days. The EDE‐A includes items reflecting two broad categories of data. The first category includes items assessing the frequency and intensity of thoughts and behaviors pertaining to weight, shape and eating patterns. These items are rated on a 7‐point scale from 0 = *no days* (i.e., characteristic was not present) to 6 = *everyday* (i.e., characteristic was present every day) and can be calculated to create four sub‐scales: restraint, eating concern, shape concern and weight concern. These sub‐scales can be summed to produce an average score ranging from 0 to 6, with higher scores indicating higher levels of ED. The second category assesses the number of days over the previous fortnight with ED behaviors (e.g., binge eating, excessive exercise, laxative use, self‐induced vomiting).The EDE‐A has been found to have acceptable internal consistency (Cronbach's alpha >.70) when used with community samples of adolescents (Mantilla et al., 2017).

#### 2.2.2 Depression

The Centre for Epidemiological Depression Scale—10 item version (CESD‐R‐10) (Andresen et al., 1994) is a 10‐item self‐report measure of depressive symptoms over the past 7 days. Items are rated on a 4‐point scale from 0 = *none of the time* (*less than 1 day*) to 3 = *most or all of the time* (*5–7 days*). An average score ranging from 0 to 30 can be calculated for each participant, with higher scores indicating higher levels of depression. The CESD‐R‐10 has been found to have excellent internal consistency (Cronbach's alpha >.85) with community samples of French adolescents (Chabrol et al., 2002) and has been validated for use with adolescent populations in the United States (Radloff, 1991).

#### 2.2.3 Health‐related quality of life

The Assessment of Quality of Life—Adolescent Instrument (AQoL‐6D) (Richardson et al., 2004) is a 20‐item measure of HRQOL over the past 7 days. The AQoL‐6D assesses HRQOL across six domains: Independent Living, Mental Health, Coping, Relationships, Pain, Senses. An AQoL‐6D total score ranging from 20 to 99 can be calculated for each participant by summing item responses, with higher scores indicating lower levels of HRQOL. A utility score may also be derived by summing weighted responses; however, utility scores were designed for use in economic evaluations and were not computed for this study. The AQoL‐6D total score was used to assess overall HRQOL (model 1) and the domain scores social relationships (model 2) and ability to cope (model 3) were used to assess the respective aspects of HRQOL (i.e., social relationships and ability to cope). The AQoL‐6D has been found to be a useful measure for assessing HRQOL in epidemiological cohort studies (Allen et al., 2013).

### 2.3 Procedure

Participants were emailed a personalized link that gave them access to an online survey containing the CESD‐R‐10, EDE‐A, and AQoL‐6D. Participants were directed to complete the survey in one allocated teaching period (i.e., school period). Trained research assistants (RAs) attended schools during the allocated teaching period to monitor the completion of the survey. Surveys were completed in student's normal classrooms or the school's gymnasium depending on teacher preference. RAs assisted participants where they had specific questions relating to survey items and/or the broader study. This process was followed at T1 and T2.

### 2.4 Statistical analysis

STATA 17 (StataCorp., 2021) was used to calculate the descriptive statistics and the main analysis was performed using Mplus 8.7 (Muthén & Muthén, 2017). Prior to the main analyses, the frequency and patterns of missing data were assessed. The frequency of missing data for continuous variables ranged from 8% to 28%. Multiple Imputation (MI) in Mplus (Rubin, 1987; Schafer, 1997), using 50 imputations, was used to address missing data. Sterne et al. (2009) suggest MI may reduce potential bias and improve statistical power when compared to analyses using complete cases. A sensitivity analysis was also performed using the full‐information maximum likelihood estimation (FIML) which uses all available data. Minimal differences between the results using MI and FIML were observed. The majority of paths that were significant remained significant and the size of coefficients were similar

**FIGURE 1:**
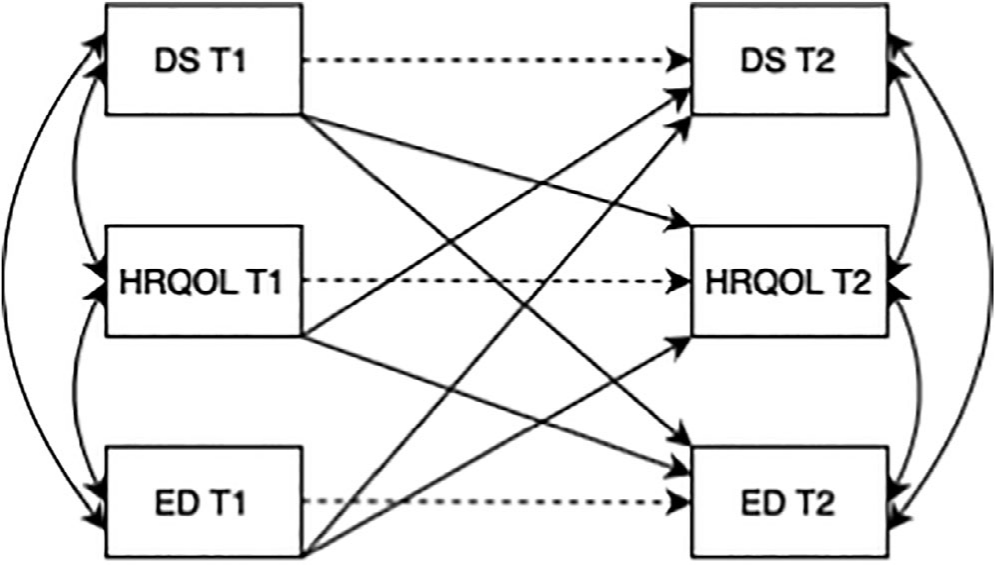
Autoregressive cross‐lagged panel model. Dotted lines indicate autoregressive paths. Straight lines indicate cross‐lag paths. Curved lines indicate covariates. DS, depressive symptoms; ED, eating disorder symptoms; HRQOL, health‐related quality of life; T1, time 1 (2019); T2, time 2 (2020).

Two‐level autoregressive cross‐lagged models with three variables (i.e., depressive symptoms, HRQOL and ED) assessed across two time points (T1 and T2) were created (see Figure 1). Multilevel autoregressive cross‐lagged models are a type of structural equation model used to assess the relationship between two or more variables over time within clustered data (i.e., students clustered within schools) (Selig & Little, 2012). These models yield three types of effects: (1) autoregressive effects, (2) cross‐lag effects and (3) correlations between two variables at the same time point (i.e., co‐variates). Autoregression effects represents the association between a variable at one time point on the same variable at the next time point (e.g., the association between depression at T1 and depression at T2) (Kearney, 2017). It can be considered the amount of stability in a construct over time. Smaller autoregressive effects indicate greater variation in the construct (Kearney, 2017). Cross‐lag effects represent the effect of one variable on another variable at a later occasion (e.g., the effect of depression at T1 on HRQOL at T2) (Selig & Little, 2012).

Multilevel cross‐lag models were required given the project data (i.e., clustered data) violates the assumption of independence. It is assumed that adolescents who attend the same school (i.e., are in the same cluster) are more likely to share similarities when compared to those attending another school (Tofighi, West & MacKinnon, 2013). Multilevel models contain within‐level (i.e., individual‐level) and between‐level (i.e., school‐level) components (Bovaird, 2007). Given this project was interested in the individual level relationships between variables, the within‐level models were of interest.

When creating multilevel models, centering predictor variables is recommended to aid the interpretation of results (Kelloway, 2015). Centering creates a distribution of scores with a mean of zero and consequently creates a common scale of measurement for the predictors (Kelloway, 2015). In this study, group level centering which involves subtracting the group mean from the individual score was applied (Kelloway, 2015). Group mean centering is recommended in situations where level 1 (i.e., individual level) effects are of interest.

Three different multilevel autoregression cross‐lagged models were created to assess the study aims. Each model was constructed using the entire sample and separately for female and male adolescents. The first model used the AQoL‐6D total score to assess HRQOL, the second used the AQoL‐6D subscale social relationships to assess the social relationships domain of HRQOL and the third model used the AQoL‐6D subscale ability to cope to assess the coping domain of HRQOL. All three models assessed depressive symptoms using the CESD‐R‐10 total score and ED symptoms using the EDE‐A total score. Standard fit indexes are not reported given they are not considered to be reliable indictors of model fit (Hsu, 2009) when estimating multilevel models and are not available when estimating multilevel models using the MLR in Mplus. An alpha level of .01 was applied to adjust for multiple testing.

## 3 RESULTS

### 3.1 Descriptive statistics

The mean, standard deviation and internal consistency for depressive symptoms, ED symptoms and HRQOL are presented in Table 1. The intraclass correlation (i.e., ratio of between‐school variance to total variance) for the outcome variables in each model are presented in the supplementary material.

**TABLE 1:** Mean, standard deviation and internal consistency for the depressive symptoms, ED symptoms and HRQOL at T1 and T2.

	Entire sample						Females						Males					
	T1		T2				T1		T2				T1		T2			
	M (*SD*)	α	M (*SD*)	α	MD (*SE*)	95% CIs	M (*SD*)	α	M (*SD*)	α	MD (*SE*)	95% CI	M (*SD*)	α	M (*SD*)	α	MD (*SE*)	95% CI
CESD‐R‐10	6.19 (4.64)	.70	7.40 (5.55)	.79	1.29 (.18)	.94–1.64	6.63 (4.78)	.72	8.59 (6.12)	.83	2.30 (.28)	1.75–2.85	5.79 (4.47)	.68	6.10 (4.65)	.71	0.31 (.22)	−.12 to .74
EDE‐A	.91 (1.04)	.87	.98 (1.25)	.87	.07 (.04)	−.003 to 0.13	1.02 (1.11)	.89	1.25 (1.36)	.93	.27 (.05)	.16–.37	.81 (.97)	.86	0.68 (1.01)	.91	−.12 (.04)	−.21 to −.03
AQoL‐6D	34.83 (8.32)	.85	34.76 (9.77)	.89	−.29 (.32)	−.91 to .34	35.38 (7.90)	.85	35.92 (9.44)	.89	.62 (.41)	−.19 to 1.42	34.33 (8.67)	.87	33.40 (9.94)	.90	−1.11 (.48)	−2.05 to −.17
Relationships	4.00 (1.35)	.53	4.09 (1.50)	.60	.14 (.05)	.03 to .24	3.83 (1.12)	.53	4.10 (1.46)	.60	.27 (.07)	.14–.41	4.14 (1.43)	.59	4.07 (1.54)	.66	−.01 (.08)	−.17 to .15
Coping	6.18 (2.00)	.67	6.54 (2.24)	.74	.34 (.07)	.20–.48	6.41 (2.06)	.67	6.91 (2.17)	.74	.51 (.10)	.32–.69	5.97 (1.92)	.65	6.16 (2.25)	.72	.18 (.10)	−.03 to .38

Abbreviations: AQoL‐6D, Assessment of Quality of Life—Adolescent instrument; CESD‐R‐10, Centre for Epidemiological Depression Scale—10 item version; coping, coping subscale of the Assessment of Quality of Life—Adolescent instrument; ED, eating disorders; EDE‐A, Eating Disorder Examination‐Questionnaire—Adolescent version; HRQOL, health‐related quality of life; relationships, social relationships subscale of the assessment of quality of life—adolescent instrument; *SD*, standard deviation; T1, time 1 (2019); T2, time 2 (2020).

### 3.2 Model 1. Depressive symptoms, eating disorder symptoms and health‐related quality of life

#### 3.2.1 Entire sample

**FIGURE 2:**
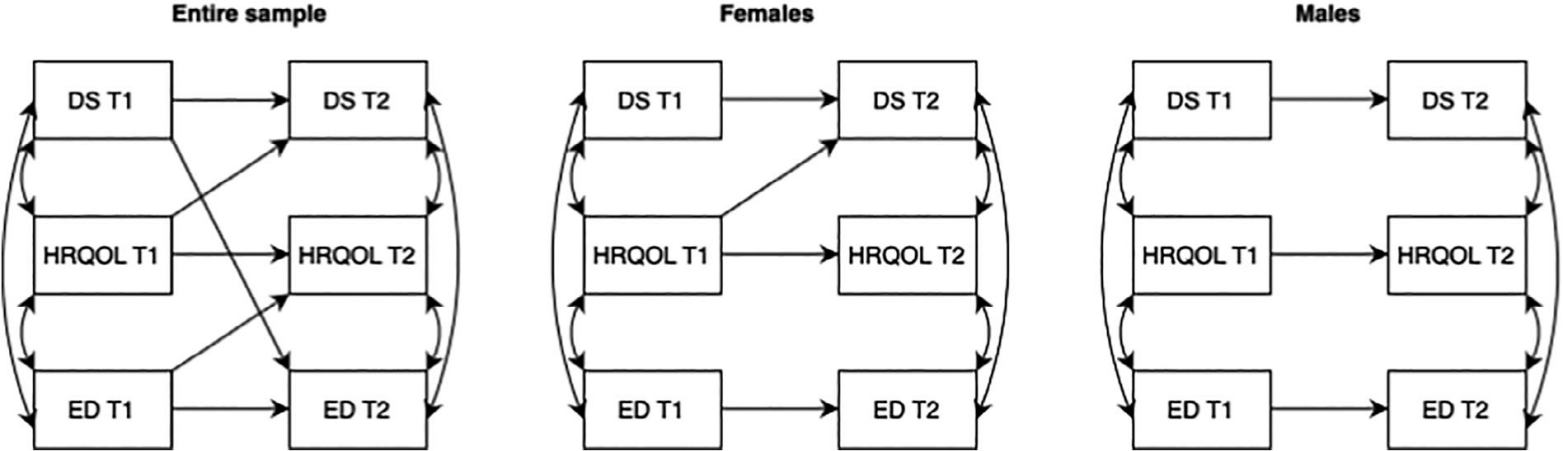
Associations between depressive symptoms, eating disorder symptoms and health‐related quality of life. Solid line indicates path is significant at the alpha level of .01. DS, depressive symptoms; ED, eating disorder symptoms; HRQOL, health‐related quality of life; T1, time 1 (2019); T2, time 2 (2020).

Model 1 assessed the reciprocal associations between depressive symptoms (CESD‐R‐10 total score), ED symptoms (EDE‐A total score) and HRQOL (AQoL‐6D total score) (Table 2). Autoregressive paths for depressive symptoms, ED symptoms and HRQOL were all significant. The cross‐lag path from HRQOL at T1 to depressive symptoms at T2 was significant. Similarly, the cross‐lag paths from ED symptoms at T1 to HRQOL at T2 was significant. Finally, the cross‐lag path between depressive symptoms at T1 and ED symptoms at T2 was significant. All significant cross‐lag associations are displayed in Figure 2.

**TABLE 2:** Standardized coefficients, standard error and 95% confidence intervals for model 1 for the entire sample.

		Entire sample			Females			Males		
		ß	95% CIs	*R* ^2^	ß	95% CIs	*R* ^2^	ß	95% CIs	*R* ^2^
DS T2	DS T1	.31	.23; .38	.23	.25	.12; .38	.27	.35	.26; .44	.22
	HRQOL T1	.19	.12; .25		.31	.19; .42		.13	.02; .23	
	ED T1	.06	−.01; .12		.001	−.08; .08		.05	−.04; .14	
HRQOL T2	DS T1	.09	.01; .17	.26	.09	−.05; .23	.32	.10	−.02; .22	.20
	HRQOL T1	.40	.33; .47		.47	.23; .71		.36	.25; .47	
	ED T1	.08	.02; .14		.06	−.03; −.15		.5	−.05; .14	
ED T2	DS T1	.11	.03; .18	.28	.11	−.02; .24	.30		.01; .21	.23
	HRQOL T1	.02	−.04; .09		.01	−.09; .11		.07	−.04; .17	
	ED T1	.47	.42; .51		.48	.38; .57		.39	.29; .49	

*Note*: *R* ^2^ indicates the explained variance for each dependent variable (i.e., depressive symptoms, HRQOL and ED symptoms at T2).

Abbreviations: ß, standardized coefficient; CI, confidence interval; DS, depressive symptoms; ED, eating disorder symptoms; HRQOL, health‐related quality of life; T1, time 1 (2019); T2, time 2 (2020).

#### 3.2.2 Females and males assessed separately

The reciprocal associations between depressive symptoms, ED symptoms and HRQOL were assessed separately for female and male adolescents (Table 2). Autoregressive paths for depressive symptoms, ED symptoms and HRQOL were all significant for both females and males. The cross‐lag path from HRQOL at T1 to depressive symptoms at T2 was significant for females. However, for males, this was only true at an alpha level of .05. All significant cross‐lag associations are displayed in Figure 2.

### 3.3 Model 2. Depressive symptoms, eating disorder symptoms and social relationships

#### 3.3.1 Entire sample

**FIGURE 3:**
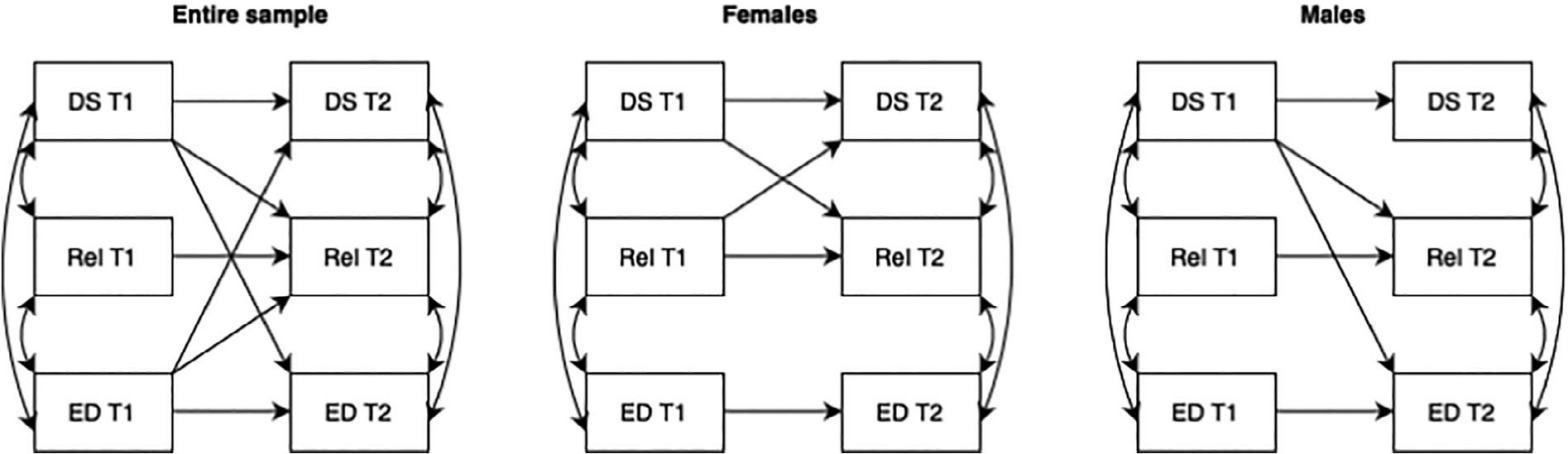
Associations between depressive symptoms, eating disorder symptoms and social relationships. Solid line indicates path is significant at the alpha level of .01. DS, depressive symptoms; ED, eating disorder symptoms; Rel, social relationships; T1, time 1 (2019), T2, time 2 (2020).

Model 2 assessed the reciprocal associations between depressive symptoms (CESD‐R‐10 total score), ED symptoms (EDE‐A total score) and social relationships (AQoL‐6D subscale relationships) (Table 3). Autoregressive paths for depressive symptoms, ED symptoms and social relationships were all significant. The cross‐lag path between ED symptoms at T1 and depressive symptoms at T2 was significant. Similarly, the cross‐lag paths between depressive symptoms and ED symptoms at T1 and social relationships at T2 were both significant. Finally, the cross‐lag path between depressive symptoms at T1 and ED symptoms at T2 was significant. All significant cross‐lag associations are displayed in Figure 3.

**TABLE 3:** Standardized coefficients, standard error and 95% confidence intervals for model 2.

		Entire sample			Females			Males		
		ß	95% CIs	*R* ^2^	ß	95% CIs	*R* ^2^	ß	95% CIs	*R* ^2^
DS T2	DS T1	.42	.35; .48	.21	.41	.32; .49	.23	.43	.35; .51	.21
	Rel T1	.01	−.07; .08		.12	.04; .20		−.01	−.11; .09	
	ED T1	.09	.02; .15		.03	−.05; .11		.07	−.02; .17	
Rel T2	DS T1	.16	.10; .22	.14	.20	.11; .30	.19	.13	.04; .22	.12
	Rels T1	.22	.16; .29		.24	.12; .35		.23	.14; .32	
	ED T1	.11	.04; .19		.13	.03; .24		.08	−.02; .17	
ED T2	DS T1	.13	.06; .19	.28	.10	−.01; .22	.30	.15	.06; .24	.23
	Rel T1	−.02	−.09; .06		.03	−.04; .11		.001	−.10; .10	
	ED T1	.47	.43; .52		.48	.32; .49		.40	.30; .51	

*Note*: *R* ^2^ indicates the proportion of variance for each dependent variable (i.e., depressive symptoms, social relationships and ED symptoms at T2).

Abbreviations: ß, standardized coefficient; CI, confidence interval; DS, depressive symptoms; ED, eating disorder symptoms; Rel, social relationships; T1, time 1 (2019); T2, time 2 (2020).

#### 3.3.2 Females and males assessed separately

The reciprocal associations between depressive symptoms, ED symptoms and social relationships were assessed separately for female and male adolescents (Table 3). Depressive symptoms at T1 predicted social relationships at T2 for both female and male adolescents. The cross‐lag path between social relationships at T1 and depressive symptoms at T2 was also significant; however, this was only true for females. Similarly, the cross‐lag path between ED symptoms at T1 and social relationships at T2 was significant for female, but not male, adolescents; this association was only significant at the alpha level of .05. Finally, the cross‐lag path between depressive symptoms at T1 and ED symptoms at T2 was significant for male, but not female, adolescents. All significant cross‐lag associations are displayed in Figure 3.

### 3.4 Model 3. Depressive symptoms, eating disorder symptoms and coping

#### 3.4.1 Entire sample

**FIGURE 4:**
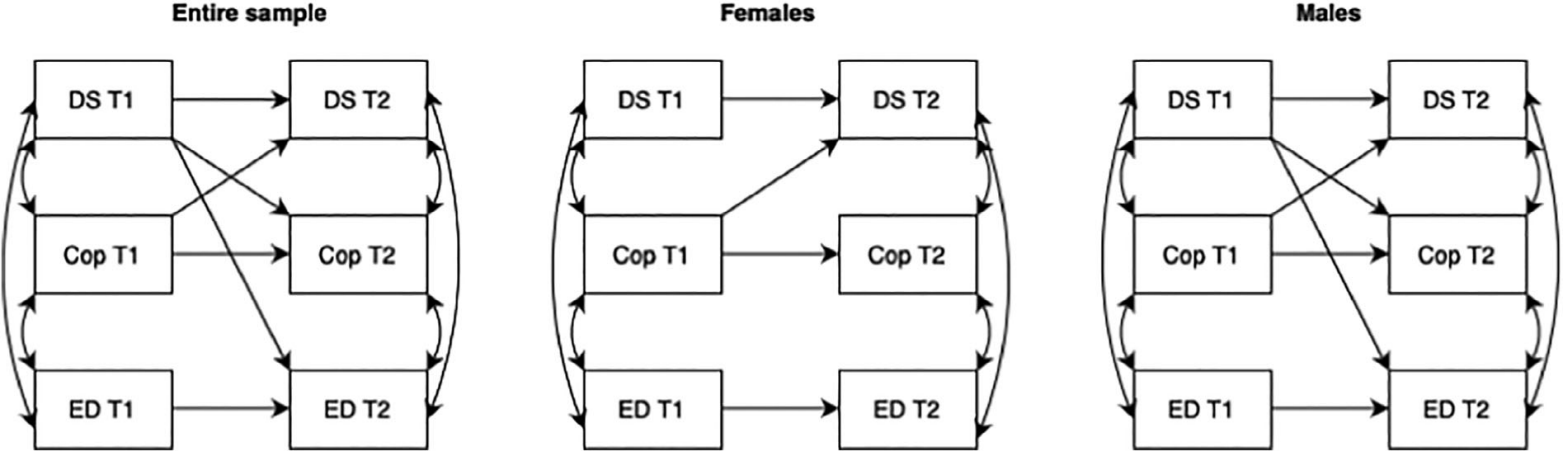
Associations between depressive symptoms, eating disorder symptoms and coping. Solid line indicates path is significant at the alpha level of .01. Cop, coping, figure does not show autoregressive paths or covariates; DS, depressive symptoms; ED, eating disorder symptoms; T1, time 1 (2019); T2, time 2 (2020).

Model 3 assessed the reciprocal associations between depressive symptoms (CESD‐R‐10 total score), ED symptoms (EDE‐A total score) and coping (AQoL‐6D subscale coping) (Table 4). Depressive symptoms at T1 predicted coping at T2 and coping at T1 predicted depressive symptoms at T2 indicating depressive symptoms and coping share a reciprocal relationship. Additionally, depressive symptoms at T1 predicted ED symptoms at T2. All significant cross‐lag associations are displayed in Figure 4.

**TABLE 4:** Standardized coefficients, standard error and 95% confidence intervals for model 3.

		Entire sample			Females			Males		
		ß	95% CIs	*R* ^2^	ß	95% CIs	*R* ^2^	ß	95% CIs	*R* ^2^
DS T2	DS T1	.33	.26; .39	.24	.33	.23; .43	.25	.37	.29; .45	.22
	Coping T1	.20	.14; .26		.22	.14; .31		.14	.05; .23	
	ED T1	.06	.01; .12		.03	−.05; .10		.05	−.03; .14	
Coping T2	DS T1	.13	.06; .20	.22	.13	−.001; .25	.27	.15	.06; .25	.16
	Coping T1	.38	.32; .44		.42	−.33; .50		.32	.22; .41	
	ED T1	.03	−.03; .08		.04	−.05; .13		−.04	−.12; .05	
ED T2	DS T1	.10	.04; .16	.28	.11	−.004; .22	.30	.14	.06; .22	.23
	Coping T1	.04	−.01; .90		.02	−.08; .11		.03	−.04; .10	
	ED T1	.47	.42; .51		.48	.38; .57		.40	.30; .50	

*Note*: *R* ^2^ indicates the proportion of variance for each dependent variable (i.e., depressive symptoms, coping and ED symptoms at T2).

Abbreviations: ß, standardized coefficient; CI, confidence interval; DS, depressive symptoms; ED, eating disorder symptoms; T1, time 1 (2019); T2, time 2 (2020).

#### 3.4.2 Females and males assessed separately

The reciprocal associations between depressive symptoms, ED symptoms and coping were assessed separately for female and male adolescents (Table 4). Coping at T1 predicted depressive symptoms at T2 for both females and males. No other cross‐lag paths were significant for female adolescents. For male adolescents, depressive symptoms at T1 predicted coping and ED symptoms at T2. All significant cross‐lag associations are displayed in Figure 4.

## 4 DISCUSSION

This exploratory study sought to assess the bi‐directional relationships between depressive symptoms, ED symptoms and HRQOL in a large sample of Australian adolescents. Findings demonstrated that HRQOL at baseline predicted depressive symptoms at the 12‐month follow‐up; this was true for the entire sample and also when females were assessed separately. However, HRQOL only predicted depressive symptoms for males at the alpha level of .05. This finding is important as it suggests decreases in HRQOL precede increases in depressive symptoms for young adolescents and thereby provides insight into the directionality of this relationship. In doing so, these findings extend studies that have reported a cross‐sectional association between depressive symptoms and HRQOL (e.g., Freire & Ferreira, 2018; Shin et al., 2022). Findings also demonstrate that ED symptoms at T1 predicted HRQOL at T2 suggesting an increase in ED symptoms results in reduced HRQOL; however, this association was not significant when females and males were assessed separately. This finding partially supports studies that have found ED symptoms to predict reduced HRQOL in community samples of adolescents (Mitchell & Steele, 2017) and young females (Wade et al., 2012).

This study also sought to assess the bi‐directional relationships between depressive symptoms, ED symptoms and social relationships as measured by the AQoL‐6D subscale relationships. When assessing the entire sample, evidence was provided to demonstrate that depressive symptoms at baseline predicted social relationships and ED symptoms at the 12‐month follow‐up. Similarly, ED symptoms at baseline predicted depressive symptoms and social relationships at the 12‐month follow‐up. However, when females and males were assessed separately, the pattern of significant associations differed by sex. For females, a bi‐directional relationship between depressive symptoms and social relationships was observed. This suggests negative social relationships may be a predictor and an outcome of depressive symptoms for adolescent females. However, for males, depressive symptoms predicted social relationships, but the reverse was not true. It is suggested that this difference may be explained, at least in part, by evidence demonstrating young adolescent females are more “people oriented” and spend a greater amount of time in social activities than young adolescent males (Perry & Pauletti, 2011). Given this, adolescent females may be more likely to experience depressive symptoms as a result of negative social relationships. Additionally, for male adolescents, depressive symptoms at T1 predicted ED symptoms at T2. This suggests, when accounting for social relationships, depressive symptoms precede ED symptoms for adolescent males, but not adolescent females. Importantly, when females and males were assessed separately, a longitudinal relationship between EDs and social relationships could not be established.

The final aim of this study was to assess the bi‐directional relationships between depressive symptoms, ED symptoms and coping as measured by the AQoL‐6D subscale coping. When assessing the entire sample, depressive symptoms at T1 predicted coping at T2 and coping at T1 predicted depressive symptoms at T2. This suggests that a reduced ability to cope increases depressive symptoms and increased depressive symptoms reduce adolescents' ability to cope. When assessing females and males separately, the cross‐lag path from coping at T1 to depressive symptoms at T2 was significant for both females and males. This supports research suggesting the use of maladaptive coping strategies can increase depressive symptoms in adolescents (Horwitz et al., 2011). However, the cross‐lag path from depressive symptoms at T1 to coping at T2, whereby increased depressive symptoms predicted lower scores on the ability to cope subscale of the AQoL‐6D, was only significant for males. This suggests the relationship between depressive symptoms and coping may differ for females and males. Additionally, consistent with the findings of model two, when females and males were assessed separately, a longitudinal relationship between EDs and coping could not be established.

## 5 IMPLICATIONS

To the authors' knowledge, this is the first study to provide insights into the reciprocal associations between depressive symptoms, ED symptoms and HRQOL in a large sample of young adolescents. Preliminary evidence is provided to demonstrate that lower HRQOL precedes depressive symptoms for young adolescent females and males. This information suggests prevention and early intervention programs designed to address adolescent depression should focus on improving HRQOL. Specifically, it is suggested that programs implement strategies that will improve adolescents' ability to cope and develop social connections; the latter may be particularly beneficial for adolescent females. Furthermore, depressive symptoms consistency predicted ED symptoms for adolescent males suggesting prevention and early intervention strategies should address depressed mood in adolescent males as a means of reducing ED symptoms.

However, this study could not establish a clear pattern of associations between HRQOL and ED symptoms. Given EDs consist of a wide range of cognitive and behavioral symptoms (American Psychiatric Association, 2013), it is suggested that individual ED symptoms (e.g., body‐related ED symptoms, restrictive symptoms) may share differential relationships with HRQOL. Assessing ED symptoms using a total score may have masked potential relationships between HRQOL and individual ED symptoms. It is also important to note that the level of ED symptomatology in this sample was relatively low. It is suggested that the relationship between ED and HRQOL may be more prominent in clinical samples.

## 6 LIMITATIONS

This study is not without limitations. Firstly, depressive symptoms, ED symptoms and HRQOL were measured using self‐report data and therefore may be susceptible to social desirability responding. Future research would benefit from using structured clinical interviews to assess symptomatology. The standardized coefficients are relatively small indicating the clinical significance of these findings may be minor. Additionally, it must be noted that differences between the models created for females and males separately could be explained by a loss of statistical power as a result of splitting the sample by sex. It is suggested that future studies recruit a large sample and conduct multigroup analysis to provide a more comprehensive understanding of sex differences. Given this, the authors recommend viewing the results as exploratory and exercising caution when interpretating study findings. Nonetheless, given this is one of the earliest studies to assess the reciprocal relationships between depressive symptoms, HRQOL and ED symptoms, the evidence provided is important to guide the development of future studies.

Furthermore, IRSD scores indicate adolescents had relatively low levels of socio‐economic disadvantage, as such, the findings may not be generalizable to adolescents across the social gradient. Despite the historical belief that EDs are more prevalent in affluent populations, a recent Australian study found ED symptoms were distributed equally across levels of socioeconomic status in a population‐based sample (Mulders‐Jones et al., 2017). Future studies would benefit from recruiting a more balanced socio‐economic sample or by weighting data to be more representative of the wider population. Finally, the models used did not include possible confounding variables (e.g., socioeconomic status, cultural background) that may have influenced findings; this should be addressed by future studies.

## 7 CONCLUSION

In a large sample of Australian adolescents, this exploratory study provided evidence to demonstrate that reduced HRQOL precedes the development of depressive symptoms and in doing so, provides a target for prevention and early intervention programs. However, when females and males were assessed separately, evidence for a longitudinal relationship between HRQOL and ED symptoms could not be established. It is suggested that future research should assess the relationship between HRQOL and individual ED symptoms (e.g., body‐related ED symptoms, restrictive symptoms) as a means of exploring relationships that may have been masked by assessing ED symptoms using a total score.

## AUTHOR CONTRIBUTIONS

**Bridget Kenny:** Conceptualization; formal analysis; project administration; writing – original draft; writing – review and editing. **Steven J. Bowe:** Formal analysis; writing – review and editing. **C. Barr Taylor:** Conceptualization; funding acquisition; writing – review and editing. **Marj Moodie:** Conceptualization; funding acquisition; supervision; writing – review and editing. **Victoria Brown:** Supervision; writing – review and editing. **Elizabeth Hoban:** Supervision; writing – review and editing. **Joanne Williams:** Conceptualization; funding acquisition; methodology; supervision.

## FUNDING INFORMATION

This study used data collected by the Supporting Healthy Image, Nutrition and Exercise (SHINE) study. The SHINE study was funded by a National Health and Medical Research Council project Grant (1122840).

## CONFLICT OF INTEREST STATEMENT

The authors have no conflicts of interest to declare.

## ETHICS STATEMENT

This project was approved by the Deakin University Human Research Ethics Committee (2019–456).

## Supporting information


**Data S1:** Supporting Information

## Data Availability

Due to the nature of this research, participants of this study did not agree for their data to be shared publicly so supporting data are not available.

## References

[eat23928-bib-0001] Allen, J. , Inder, K. J. , Lewin, T. J. , Attia, J. R. , & Kelly, B. J. (2013). Contruct validity of the assessment of quality of life—6D (AQoL‐6D) in community samples. Health and Quality of Life Outcomes, 11(61), 61. 10.1186/1477-7525-11-61 23590808 PMC3639231

[eat23928-bib-0002] American Psychiatric Association . (2013). Diagnostic and statistical manual of mental disorders (5th ed.). American Psychiatric Association.

[eat23928-bib-0003] Andresen, E. M. , Malmgren, J. A. , Carter, W. B. , & Patrick, D. L. (1994). Screening for depression in well older adults: Evaluation of a short form of the CES‐D. American Journal of Preventive Medicine, 10(2), 77–84. 10.1016/S0749-3797(18)30622-6 8037935

[eat23928-bib-0004] Australian Bureau of Statistics . (2011). *Socio‐economic indexes for areas* (*SEIFA*). Canberra, Australia. Retrieved from https://www.ausstats.abs.gov.au/ausstats/subscriber.nsf/0/22CEDA8038AF7A0DCA257B3B00116E34/$File/2033.0.55.001%20seifa%202011%20technical%20paper.pdf

[eat23928-bib-0005] Bovaird, J. A. (2007). Multilevel structural models for contextual factors. In T. D. Little , J. A. Bovaird , & N. A. Card (Eds.), Modelling contextual effects in longitudinal data. Erlbaum.

[eat23928-bib-0006] Carter, J. C. , Stewart, D. A. , & Fairburn, C. G. (2001). Eating disorder examination questionnaire: Norms for young adolescent girls. Behaviour Research and Therapy, 39(5), 625–632. 10.1016/S0005-7967(00)00033-4 11341255

[eat23928-bib-0007] Chabrol, H. , Montovany, A. , Chouicha, K. , & Duconge, E. (2002). Study of the CES‐D on a sample of 1,953 adolescent students. L'encéphale, 28(5 Pt 1), 429–432. 12386544

[eat23928-bib-0008] Crockett, M. A. , Martínez, V. , & Jiménez‐Molina, Á. (2020). Subthreshold depression in adolescence: Gender differences in prevalence, clinical features, and associated factors. Journal of Affective Disorders, 272, 269–276. 10.1016/j.jad.2020.03.111 32553367

[eat23928-bib-0009] Fairburn, C. (2008). Cognitive behavior therapy and eating disorders. Guilford Publications.

[eat23928-bib-0010] Freire, T. , & Ferreira, G. (2018). Health‐related quality of life of adolescents: Relations with positive and negative psychological dimensions. International Journal of Adolescence and Youth, 23(1), 11–24. 10.1080/02673843.2016.1262268

[eat23928-bib-0011] Goldschmidt, A. B. , Wall, M. , Choo, T. H. , Becker, C. , & Neumark‐Sztainer, D. (2016). Shared risk factors for mood‐, eating‐, and weight‐related health outcomes. Health Psychology, 35(3), 245–252. 10.1037/hea0000283 26690639 PMC4760867

[eat23928-bib-0012] Hellström, L. , & Beckman, L. (2021). Life challenges and barriers to help seeking: Adolescents' and young adults' voices of mental health. International Journal of Environmental Research and Public Health, 18(24), 13101. 10.3390/ijerph182413101 34948711 PMC8700979

[eat23928-bib-0013] Horwitz, A. G. , Hill, R. M. , & King, C. A. (2011). Specific coping behaviors in relation to adolescent depression and suicidal ideation. Journal of Adolescence, 34(5), 1077–1085. 10.1016/j.adolescence.2010.10.004 21074841 PMC3319342

[eat23928-bib-0014] Hsu, H.‐Y. (2009). Testing the effectiveness of various commonly used fit indices for detecting misspecifications in multilevel structural equation models. (Doctor of Philosophy). Texas A&M University Retrieved from https://methods.sagepub.com/reference/the‐sage‐encyclopedia‐of‐communication‐research‐methods

[eat23928-bib-0015] Hughes, E. , Goldschmidt, A. , Labuschagme, Z. , Loeb, K. , Sawyer, S. , & Le Grange, D. (2013). Eating disorders with and without comorbid depresion and anxiety: Similiarities and differences in a clinical sample of children and adolescents. Eating Disorders Review, 21(5), 386–394. 10.1002/erv.2234 23681932

[eat23928-bib-0016] Im, Y. , Oh, W.‐O. , & Suk, M. (2017). Risk factors for suicide ideation among adolescents: Five‐year national data analysis. Archives of Psychiatric Nursing, 31(3), 282–286. 10.1016/j.apnu.2017.01.001 28499568

[eat23928-bib-0017] Jalali‐Farahani, S. , Chin, Y. , Mohd Nasir, M. , & Amiri, P. (2015). Disordered eating and its association with overweight and health‐related quality of life among adolescents in selected high schools of Tehran. Child Psychiatry & Human Development, 46(3), 485–492. 10.1007/s10578-014-0489-8 25173517

[eat23928-bib-0018] Jenkins, P. E. , Hoste, R. R. , Doyle, A. C. , Eddy, K. , Crosby, R. D. , Hill, L. , Powers, P. , Mitchell, J. E. , & Le Grange, D. (2014). Health‐related quality of life among adolescents with eating disorders. Journal of Psychosomatic Research, 76(1), 1–5. 10.1016/j.jpsychores.2013.11.006 24360133

[eat23928-bib-0019] Johnson, D. , Dupuis, G. , Piche, J. , Clayborne, Z. , & Colman, I. (2018). Adult mental health outcomes of adolescent depression: A systematic review. Depression and Anxiety, 35(8), 700–716. 10.1002/da.22777 29878410

[eat23928-bib-0020] Karkkainen, U. , Mustelin, L. , Raevuori, A. , Kaprio, J. , & Keski‐Rahkonen, A. (2017). Do disordered eating behaviours have long‐term health consequences? European Eating Disorders Review, 26(1), 22–28. 10.1002/erv.2568 29160017 PMC5732059

[eat23928-bib-0021] Kearney, M. W. (2017). Cross‐lagged panel analysis. In M. R. Allen (Ed.), Sage encyclopedia of communication research methods. SAGE Publishing.

[eat23928-bib-0022] Kelloway, E. K. (2015). Using Mplus. In Using Mplus for structural equation modeling: A Researcher's guide (2nd ed.). SAGE Publications. 10.4135/9781483381664

[eat23928-bib-0023] Kenny, B. , Fuller‐Tyszkiewicz, M. , Moodie, M. , Brown, V. , & Williams, J. (2022). Bi‐directional associations between depressive symptoms and eating disorder symptoms in early adolescence. Body Image, 42, 246–256. 10.1016/j.bodyim.2022.06.012 35841698

[eat23928-bib-0024] Kinasz, K. , Accurso, E. C. , Kass, A. E. , & Le Grange, D. (2016). Does sex matter in the clinical presentation of eating disorders in youth? The Journal of Adolescent Health, 58(4), 410–416. 10.1016/j.jadohealth.2015.11.005 26830976 PMC4808325

[eat23928-bib-0025] Koushiou, M. , Nikolaou, P. , & Karekla, M. (2019). Prevalence and correlates of eating disorders in Greek‐Cypriot adolescents and young adults. European Journal of Counselling Psychology, 8(1), 3–18. 10.5964/ejcop.v8i1.170

[eat23928-bib-0026] Lawrence, D. , Johnson, S. , Hafekost, J. , Boterhoven de Haan, K. , Sawyer, M. , Ainley, J. , & Zubrick, S. R. (2015). The mental health of children and adolecents: Report on the second Australian child and adolescent survey of mental health and wellbeing. Australian Government Department of Health and Aged Care. Retrieved from Canberra, Australia.

[eat23928-bib-0027] Long, E. , Gardani, M. , McCann, M. , Sweeting, H. , Tranmer, M. , & Moore, L. (2020). Mental health disorders and adolescent peer relationships. Social Science & Medicine, 253, 112973. 10.1016/j.socscimed.2020.112973 32283352 PMC7248572

[eat23928-bib-0028] Mantilla, E. , Birgegård, A. , & Clinton, D. (2017). Factor analysis of the adolescent version of the eating disorders examination questionnaire (EDE‐Q): Results from Swedish general population and clinical samples. Journal of Eating Disorders, 5, 1–8 Retrieved from https://search.ebscohost.com/login.aspx?direct=true&db=edb&AN=123413979&authtype=sso&custid=deakin&site=eds‐live&scope=site 28580141 10.1186/s40337-017-0140-8 PMC5452401

[eat23928-bib-0029] McCabe, M. , & Ricciardelli, L. (2006). A prospective study of extreme weight change behaviors among adolescent boys and girls. Journal of Youth and Adolescence, 35(3), 425–434. 10.1007/s10964-006-9062-5

[eat23928-bib-0030] McGuinness, T. M. , Dyer, J. G. , & Wade, E. H. (2012). Gender differences in adolescent depression. Journal of Psychosocial Nursing and Mental Health Services, 50(12), 17–20. 10.3928/02793695-20121107-04 23457713

[eat23928-bib-0031] Mitchell, T. B. , & Steele, R. G. (2017). Bidirectional associations between disordered eating and health‐related quality of life in elementary school‐age youth. Journal of Pediatric Psychology, 42(3), 315–324. 10.1093/jpepsy/jsw082 27771616

[eat23928-bib-0032] Mulders‐Jones, B. , Mitchison, D. , Girosi, F. , & Hay, P. (2017). Socioeconomic correlates of eating disorder symptoms in an Australian population‐based sample. PLoS One, 12(1), e0170603. 10.1371/journal.pone.0170603 28141807 PMC5283666

[eat23928-bib-0033] Muthén, L. K. , & Muthén, B. O. (2017). Mplus User's Guide (8th ed.). Muthén & Muthén.

[eat23928-bib-0034] Neumark‐Sztainer, D. , Wall, M. , Larson, N. I. , Eisenberg, M. E. , & Loth, K. (2011). Dieting and disordered eating behaviors from adolescence to young adulthood: Findings from a 10‐year longitudinal study. Journal of the American Dietetic Association, 111(7), 1004–1011. 10.1016/j.jada.2011.04.012 21703378 PMC3140795

[eat23928-bib-0035] Orben, A. , Tomova, L. , & Blakemore, S. J. (2020). The effects of social deprivation on adolescent development and mental health. The Lancet Child and Adolescent Health, 4(8), 634–640. 10.1016/s2352-4642(20)30186-3 32540024 PMC7292584

[eat23928-bib-0036] Perry, D. G. , & Pauletti, R. E. (2011). Gender and adolescent development. Journal of Research on Adolescence, 21, 61–74. 10.1111/j.1532-7795.2010.00715.x

[eat23928-bib-0037] Radloff, L. (1991). The use of the Centre for Epidemiological Studies Depression Scale in adolescents and young adults. Journal of Youth and Adolescence, 20(2), 146–166. 10.1007/BF01537606 24265004

[eat23928-bib-0038] Revicki, D. A. , Kleinman, L. , & Cella, D. (2014). A history of health‐related quality of life outcomes in psychiatry. Dialogues in Clinical Neuroscience, 16(2), 127–135. 10.31887/DCNS.2014.16.2/drevicki 25152652 PMC4140507

[eat23928-bib-0039] Richardson, J. R. J. , Day, N. A. , Peacock, S. J. , & Iezzi, A. (2004). Measurement of the quality of life for economic evaluation and the assessment of quality of life (AQoL) mark 2 instrument. Australia Economic Review (Vol. 37, 62‐88). Blackwell Publishing Asia.

[eat23928-bib-0040] Rubin, D. B. (1987). Multiple imputation for nonresponse in surveys (Vol. 81). John Wiley & Sons.

[eat23928-bib-0041] Saeedzadeh Sardahaee, F. , Lingaas Holmen, T. , Micali, N. , & Kvaløy, K. (2017). Suicidal ideation amongst adolescent suffering from disordered eating: The young‐HUNT study. European Psychiatry, 41(S1), S88–S89. 10.1016/j.eurpsy.2017.01.278

[eat23928-bib-0042] Sander, J. , Moessner, M. , & Bauer, S. (2021). Depression, anxiety and eating disorder‐related impairment: Moderators in female adolescents and young adults. International Journal of Environmental Research and Public Health, 18(5), 2779. 10.3390/ijerph18052779 33803367 PMC7967486

[eat23928-bib-0043] Schafer, J. L. (1997). Analysis of incomplete multivariate data. CRC Press.

[eat23928-bib-0044] Schaumberg, K. , Welch, E. , Breithaupt, L. , Hübel, C. , Baker, J. H. , Munn‐Chernoff, M. A. , Yilmaz, Z. , Ehrlich, S. , Mustelin, L. , Ghaderi, A. , Hardaway, A. J. , Bulik‐Sullivan, E. C. , Hedman, A. M. , Jangmo, A. , Nilsson, I. A. K. , Wiklund, C. , Yao, S. , Seidel, M. , & Bulik, C. M. (2017). The science behind the academy for eating disorders' nine truths about eating disorders. European Eating Disorders Review, 25(6), 432–450. 10.1002/erv.2553 28967161 PMC5711426

[eat23928-bib-0045] Selig, J. P. , & Little, T. D. (2012). Autoregressive and cross‐lagged panel analysis for longitudinal data. In B. Laursen , T. D. Little , & N. A. Card (Eds.), Handbook of developmental research methods. Guilford Press.

[eat23928-bib-0046] Shin, H. , Jeon, S. , & Cho, I. (2022). Factors influencing health‐related quality of life in adolescent girls: A path analysis using a multi‐mediation model. Health and Quality of Life Outcomes, 20(1), 50. 10.1186/s12955-022-01954-6 35331239 PMC8943919

[eat23928-bib-0047] Shorey, S. , Ng, E. D. , & Wong, C. H. J. (2022). Global prevalence of depression and elevated depressive symptoms among adolescents: A systematic review and meta‐analysis. British Journal of Clinical Psychology, 61(2), 287–305. 10.1111/bjc.12333 34569066

[eat23928-bib-0048] Sparti, C. , Santomauro, D. , Cruwys, T. , Burgess, P. , & Harris, M. (2019). Disordered eating among Australian adolescents: Prevalence, functioning, and help received. International Journal of Eating Disorders, 52(3), 246–254. 10.1002/eat.23032 30734332

[eat23928-bib-0049] StataCorp. (2021). Stata statistical software: Release 17. StataCorp LLC.

[eat23928-bib-0050] Sterne, J. A. , White, I. R. , Carlin, J. B. , Spratt, M. , Royston, P. , Kenward, M. G. , Wood, A. M. , & Carpenter, J. R. (2009). Multiple imputation for missing data in epidemiological and clinical research: Potential and pitfalls. BMJ, 338, b2393. 10.1136/bmj.b2393 19564179 PMC2714692

[eat23928-bib-0051] Tang, X. , Tang, S. , Ren, Z. , & Wong, D. F. K. (2019). Prevalence of depressive symptoms among adolescents in secondary school in mainland China: A systematic review and meta‐analysis. Journal of Affective Disorders, 245, 498–507. 10.1016/j.jad.2018.11.043 30439677

[eat23928-bib-0052] Thapar, A. , Collishaw, S. , Pine, D. S. , & Thapar, A. K. (2012). Depression in adolescence. Lancet, 379(9820), 1056–1067. 10.1016/S0140-6736(11)60871-4 22305766 PMC3488279

[eat23928-bib-1001] Tofighi, D. , West, S. G. , & Mackinnon, D. P. (2013). Multilevel mediation analysis: The effects of omitted variables in the 1‐1‐1 model. The British journal of mathematical and statistical psychology, 66(2), 290–307. https://10.1111/j.2044-8317.2012.02051.x 22594884 10.1111/j.2044-8317.2012.02051.x PMC4814716

[eat23928-bib-0053] Touchette, E. , Henegar, A. , Godart, N. , Pryor, L. , Falissard, B. , Tremblay, R. , & Côté, S. (2011). Subclinical eating disorders and their comorbidity with mood and anxiety disorders in adolescent girls. Psychiatry Research, 185(1‐2), 185–192. 10.1016/j.psychres.2010.04.005 20546924

[eat23928-bib-0054] Volpe, U. , Tortorella, A. , Manchia, M. , Monteleone, A. M. , Albert, U. , & Monteleone, P. (2016). Eating disorders: What age at onset? Psychiatry Research, 238, 225–227. 10.1016/j.psychres.2016.02.048 27086237

[eat23928-bib-0055] Wade, T. D. , Wilksch, S. M. , & Lee, C. (2012). A longitudinal investigation of the impact of disordered eating on young women's quality of life. Health Psychology, 31(3), 352–359. 10.1037/a0025956 22059619

[eat23928-bib-0056] Zervaki, K. , Yiannakouris, N. , Sdrali, D. , & Costarelli, V. (2017). Diet quality, disordered eating and health‐related quality of life in Greek adolescents. Nutrition & Food Science, 47(4), 511–521. 10.1108/NFS-12-2016-0189

