# Supplementary material for: Longitudinal relationships between sub‐clinical depression, sub‐clinical eating disorders and health‐related quality of life in early adolescence
Source: Int J Eat Disord. 2023 Mar 9;56(6):1114–24. doi: 10.1002/eat.23928 (PMC10946984; doi:10.1002/eat.23928)
Supplement: Supplementary file 1 — Data S1: Supporting Information [file EAT-56-1114-s001.docx]

**Longitudinal relationships between sub-clinical depression, sub-clinical eating disorders and health-related quality of life in early adolescence**

Supplementary material

|  |  | Entire sample | Females | Males |
| --- | --- | --- | --- | --- |
| Model 1 | Depression | 0.05 | 0.05 | 0.02 |
|  | HRQOL | 0.03 | 0.03 | 0.01 |
|  | EDs | 0.03 | 0.02 | 0.04 |
|  |  |  |  |  |
| Model 2 | Depression | 0.05 | 0.05 | 0.02 |
|  | Relationships | 0.04 | 0.02 | 0.05 |
|  | EDs | 0.04 | 0.02 | 0.04 |
|  |  |  |  |  |
| Model 3 | Depression | 0.05 | 0.05 | 0.03 |
|  | Coping | 0.01 | 0.03 | 0.00 |
|  | EDs | 0.04 | 0.02 | 0.03 |

Table 1. Intraclass correlation coefficient for dependent variables

Note. ED = eating disorders, HRQOL = health-related quality of life
